# Validation of the Arabic version of the “Cognitive Theory of Mind (ToM-15) scale” among Lebanese patients with schizophrenia^☆^

**DOI:** 10.1016/j.scog.2026.100442

**Published:** 2026-05-08

**Authors:** Maha Bteich, Carole Kassamany, Chadia Haddad, Hala Sacre, Benjamin Calvet, Pascale Salameh

**Affiliations:** a Faculty of Public Health, Lebanese University, Fanar, Lebanon; b Psychiatric Hospital of the Cross, Jal Eddib, Lebanon; c INSPECT-LB (Institut National de Santé Publique, d'Épidémiologie Clinique et de Toxicologie-Liban), Beirut, Lebanon; d Inserm U1094, IRD UMR270, Univ. Limoges, CHU Limoges, EpiMaCT - Epidemiology of Chronic Diseases in Tropical Zone, Institute of Epidemiology and Global Health– Michel Dumas, OmegaHealth, Limoges, France; e Pôle Universitaire de Psychiatrie de l'Adulte et de la Personne Âgée, d'Addictologie, Centre Hospitalier Esquirol, 87000, Limoges, France; f Unité de Recherche et Innovation, Centre Hospitalier Esquirol, 87000, Limoges, France; g School of Medicine, Lebanese American University, Byblos, Lebanon; h Faculty of Pharmacy, Lebanese University, Hadat, Lebanon; i Department of Primary Care and Population Health, University of Nicosia Medical School, 2417, Nicosia, Cyprus

**Keywords:** Schizophrenia, theory of mind, Cognitive assessment, Validation study, Lebanese population

## Abstract

**Background:**

Schizophrenia is characterized by cognitive and social impairments, including deficiencies in Theory of Mind (ToM). Although numerous ToM assessment instruments are available, none have been validated for Lebanese Arabic-speaking individuals with schizophrenia. This study aimed to translate and validate the Arabic version of the Cognitive Theory of Mind Scale (ToM-15).

**Methods:**

A cross-sectional study was performed at the Psychiatric Hospital of the Cross in Lebanon from March to June 2025. The sample consisted of 75 clinically stable in-patients diagnosed with schizophrenia and 50 healthy participants. Participants administered the Arabic ToM-15 in conjunction with other validated instruments evaluating social and cognitive functioning.

**Results:**

Patients with schizophrenia exhibited significantly lower scores on all ToM-15 assessments compared to healthy participants (Total ToM-15 score: 18.94 ± 0.65 vs. 27.11 ± 0.85; *p* < 0.001). The Arabic ToM-15 demonstrated a good internal consistency (Cronbach's α = 0.817) and a five-factor structure accounting for 51.8% of the variance (KMO = 0.430; Bartlett's test <0.001). A positive correlation was identified between the total score of the ToM-15 and its subscales (*r* = 0.929 and *r* = 0.737; *p* ≤ 0.001). A negative correlation was identified between ToM-15 total scores and cognitive complaint scores (*r* = −0.266, *p* < 0.05), while no correlation was detected with social cognitive complaint and empathy.

**Conclusion:**

The Arabic ToM-15 demonstrates acceptable reliability and preliminary evidence of validity for evaluating cognitive Theory of Mind in patients with schizophrenia. Further studies with larger and more diverse samples is required to support these findings.

## 1 Introduction

Schizophrenia, a severe mental illness that affects 1% of the global population, is more prevalent in men, and symptoms typically manifest in late adolescence or early adulthood. Genetic, environmental, and neurobiological factors are among the causes (Hany and A., 2024). In Lebanon, schizophrenia is estimated to afflict approximately 50,000 individuals in a population of approximately five million. However, precise local epidemiological data—particularly in the aftermath of recent crises—are still sparse (Kassir et al., 2022). Schizophrenia is characterized by cognitive deficits, which impact up to 75% of patients who typically experience impairments in numerous cognitive functions, including memory, attention, executive function, motor skills, and social cognition (Millan et al., 2012).

Among the functions affected by schizophrenia are deficits in theory of mind (ToM). The ToM enables one to contemplate the thoughts of others and to reason about what they believe, intend, or perceive (Duval et al., 2011). The First-order false belief has been the most prevalent subject in ToM research, which is the recognition that it is feasible to harbor deceptive beliefs regarding events in the world. The Second-order false belief is a more sophisticated development, which involves the recognition that it is feasible to harbor a false belief regarding the beliefs of another individual **(****Miller, 2009****)**. The deficits at the level of the ToM have been consistently associated with adverse functional and clinical outcomes, underscoring the critical significance of assessing ToM to understand and address these impairments in schizophrenia (van Neerven et al., 2021). As a result, ToM is a prospective target for intervention strategies that are designed to improve the functional outcomes of patients with schizophrenia (de Sales et al., 2023).

ToM is typically evaluated using false-belief tests, such as the Sally–Anne Test (Wimmer and Perner, 1983). Other paradigms encompass deception tests (Perrotta, 2020), Baron-Cohen's Reading the Mind in the Eyes Test (Baron-Cohen et al., 2001), and the Picture Sequencing Task (Brüne et al., 2009). More extensive instruments, such as the Edinburgh Social Cognition Test, the Awareness of Social Inference Test–Short Form and the Faux Pas Test assess both cognitive and affective dimensions of ToM (Baksh et al., 2018; Honan et al., 2016; Giannakou et al., 2019). The Strange Stories task developed by Happé in 1994, evaluates the capacity to infer mental states in oneself and others, which is a component of ToM. This tool has been subjected to translations, revisions, and adaptations since that time (Happé, 1994).

Another test specifically designed to assess cognitive ToM was developed by Béatrice Desgranges and colleagues in 2012. The ToM-15 task evaluates objectively the individual's capacity to discern and comprehend the mental states of others. Instead of relying on the examiner's opinion or general observation, the tool provides specific, structured questions with clear scoring criteria. The original study was done among 175 healthy subjects aged 20 to 90 years, providing data across five age groups and two educational levels. These findings underscore its utility in assessing ToM across a broad demographic spectrum. It consists of 15 narratives, including 8 first-order and 7 second-order false belief stories. The task is divided into two components: a false belief task and a comprehension task (Desgranges et al., 2012).

In the Arab countries, the field of ToM research is expanding, with particular emphasis on the impact of language and culture. However, it has primarily concentrated on children with autism spectrum disorder rather than schizophrenia (Jelili et al., 2022; Abd El-Raziq et al., 2025). Some tools have been translated into Arabic and used with normally-developing and neurotypical children in several Arabic-speaking countries (Moawad, 2017; Rajhi et al., 2020; Salhi et al., 2023). In the clinical domain, a study conducted at the Okasha Institute of Psychiatry, Ain Shams University Hospital (Egypt) compared the performance of ToM in patients with acute psychosis, schizophrenia, and healthy controls using the Reading the Mind in the Eyes Test, an advanced measure of ToM. The results showed that patients with schizophrenia exhibited substantially poorer ToM performance than those with acute psychosis, and both patient groups exhibited impairments in comparison to healthy controls (El Ghamry et al., 2022).

Few psychometric scales and clinical instruments have been validated for the assessment of social cognitive function among patients with schizophrenia in Lebanon (Haddad et al., 2023; Haddad et al., 2021a; Haddad et al., 2022). The Assessment of the Arabic Social Cognition Scale (ACSo) (Haddad et al., 2026) is used to assess subjective complaints pertaining to social cognition across four domains: theory of mind, emotional processing, social perception and knowledge, and attributional biases. Nevertheless, ACSo lacks a direct assessment of Theory of Mind and depends mainly on self-reported, subjective assessments rather than objective performance-based measures (Graux et al., 2019). In addition, the Arabic OSCARS may assist clinicians and researchers in Arab contexts by offering a standardized observational tool, thereby addressing the existing deficiency in social cognition evaluation in schizophrenia (Fekih-Romdhane et al., 2025). This device is regarded as highly beneficial for clinical practice and research applications due to its ease and quickness of administration. However, while external evaluations from observers (interviewers or informants) may reduce the significance of insights, additional research is necessary to investigate the psychometric properties of the OSCARS when utilized as both an informant and self-report measure, and to compare these with the OSCARS interviewer report (Healey et al., 2015).

At present, there is no instrument in Lebanon that has been translated into Arabic, validated in patients with schizophrenia, and that measures the TOM in a specific and objective manner. The absence of appropriate assessment instruments often leads clinicians to underestimate cognitive impairment in schizophrenia. Relying on untranslated or invalidated tests can lead to inaccurate evaluations and culturally irrelevant results. This underscores the pressing necessity of validating and using reliable cognitive assessment instruments that are appropriate for Arabic-speaking populations. A validated test that is culturally appropriate would benefit patients with schizophrenia by improving diagnosis, treatment follow-up, and the development of cognitive rehabilitation and social skills.

Therefore, this study aims to validate the Arabic version of the “cognitive theory of mind (ToM-15) scale” in a sample of Lebanese inpatients with schizophrenia. This will provide clinicians and researchers with a standardized instrument to evaluate theory of mind deficits in this population. Ultimately, this validation will assist in addressing the gap in locally available measures and support the better evaluation and management of cognitive and social impairments in schizophrenia.

## 2 Methods

### 2.1 Study design and participants

A cross-sectional study was conducted at the Psychiatric Hospital of the Cross (HPC)-Lebanon, between March and June 2025, that enrolled 75 chronic in-patients diagnosed with schizophrenia disorders and 50 healthy participants. Healthy participants, defined as individuals without any psychiatric disorders, were recruited from among hospital employees and the surrounding community.

The inclusion criteria were as follows: patients with schizophrenia clinically stable, aged 18 or older, had a minimum of 5 years of education, and were diagnosed with schizophrenia disorders according to the DMS-5 diagnostic criteria. Exclusion criteria included patients who were diagnosed with intellectual disability, had a neurological history, substance misuse, or other comorbidities that could potentially disrupt neuropsychological tests. In addition, patients who declined to participate in all the evaluations or who were unable to understand the instructions for the various neuropsychological tests were excluded.

### 2.2 Ethical approval

The Ethics and Research Committee at the Psychiatric Hospital of the Cross approved this study (HPC-003-10-24) in compliance with the Hospital's Regulatory Research Protocol. This study was implemented in accordance with the ethical standards of the Helsinki Declaration. We obtained written informed consent from each participant before enrollment. Participation was entirely voluntary, and no monetary or material compensation was made. Personal information that was collected was not identified, and anonymity and confidentiality were strictly maintained.

### 2.3 Sample size calculation

According to a study conducted by Comrey and Lee (2013) (Comrey and Lee, 2013), the scale validation procedure requires 5–10 observations per item. 75 patients with schizophrenia were selected to participate in the ToM-15 test, which comprises 15 stories. Five patients were required for each narrative. To guarantee comparability of the groupings, a healthy participant group was also incorporated. As a result, the research included 125 participants, including 75 patients and 50 healthy participants.

### 2.4 Translation procedure

The ToM-15 was developed in French-speaking populations (Desgranges et al., 2012). Therefore, a forward-backward translation into Arabic was done for this study, and the linguistic and conceptual equivalence was ensured by expert review. The test was first translated from French into Arabic by a translator, then two other translators who had not seen the original text translated it back into French. Finally, a comparison between the original text and the back-translated ones led to a version with alignment in meaning between the forward and backward versions. Clarity and cultural appropriateness were confirmed after a pilot test with a small group of participants who were not included in the final sample. Contact was established with the original French research group that developed the scale, confirming that permission to use the scale could be granted.

### 2.5 Data collection and measures

The sample of patients with schizophrenia was selected randomly from a list generated from the hospital's computer software. Healthy individuals were recruited from the HPC staff and the surrounding community. Two trained researchers explained the purpose of the study and administered the test to patients and healthy participants after obtaining informed consent.

The questionnaire used in this study was written in Arabic, the native language in Lebanon. The first part of the questionnaire assessed the sociodemographic characteristics of the participants, including age, gender, education level, and marital status. The second part is designated to collect data about mental health, family history of psychiatric illness, type of schizophrenia, duration of hospitalization, length of illness, and number of hospital admissions. The information related to patients was determined by reviewing their medical records.

**Fig. 1:**
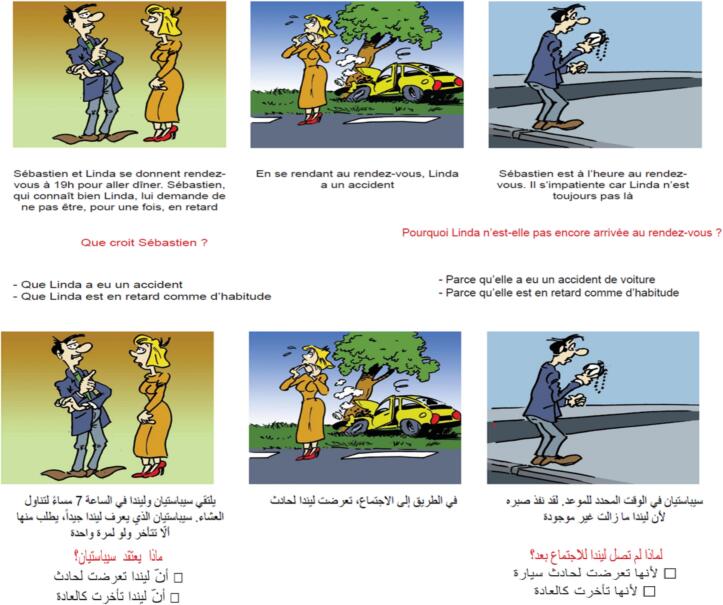
Example of a TOM-15 Story (Arabic Version) Adapted from the French Version, Including the False-Belief and Comprehension Tasks
• **ACSo - Self-Assessment of Social Cognition Impairments:** this test contains 12 items covering four domains of social cognition (Emotional Processes, Theory of Mind, Attributional Biases, Social Perception and Knowledge) (Graux et al., 2019). Each item is rated on a five-point Likert scale from most of the time, often, sometimes, rarely, to never. It is scored from 0 to 4 (5 points), with a total possible score ranging from 0 to 48. A higher score means more subjective complaints and perceived difficulties in social cognition. This scale has been validated in the Arabic language in Lebanon (Haddad et al., 2026). In the current study, the Cronbach alpha value was 0.643.
• **SASCCS – Self-Assessment Scale of Cognitive Complaints in Schizophrenia:** 21 items covering five cognitive domains (Memory, Attention, Executive functions, Language, Praxis) (Johnson et al., 2009). Each item is rated on a 5-point Likert-type scale from never, rarely, sometimes, often, and very often. It is scored from 0 to 4 (5 points), with a total possible score ranging from 0 to 84 (Johnson et al., 2009). The total score is the sum of all item scores, and a higher score means more subjective complaints and greater perceived cognitive difficulties. This scale has been validated in the Arabic language in Lebanon (Haddad et al., 2023). In the current study, the Cronbach alpha value was 0.825.
• **QCAE – Questionnaire of Cognitive and Affective empathy:** 31 items grouped into five first-order subscales, which load onto the two broader empathy dimensions (Cognitive Empathy and Affective Empathy) (Reniers et al., 2011). Items are answered using a 4-point Likert scale from strongly agree, agree, disagree, and strongly disagree, scored from 1 to 4 (4 points). The coding of certain elements should be reversed prior to the calculation of the scores. The cognitive empathy score is the sum of the Perspective Taking and Online Simulation subscales, and the affective empathy score is the sum of Emotion Contagion, Proximal Responsivity, and Peripheral Responsivity. In conclusion, the total score is determined by the sum of the cognitive and affective empathy scores. A higher score indicates more cognitive and/or affective empathy, depending on the domain (Reniers et al., 2011). In the current study, the Cronbach alpha value was 0.822.

The third part of the questionnaire included the following scales:

• **ToM-15 test:** it is a false belief test which contains fifteen stories, eight for the first order and seven for the second order (Desgranges et al., 2012). The test is divided into two components: a false belief task and a comprehension task that uses the same stories but different questions (Fig. 1). The first score is determined by the number of right answers to the first-order questions (up to 8), and the second score is determined by the number of right answers to the second-order questions (up to 7). The fifteen stories are presented to the participant one more time once the false belief task is finished, and they must respond to a question for each story to demonstrate understanding. The total score of the false belief task (ToM-15 score 1, up to 15) was calculated by summing up the number of correct responses and the same was done to get the total score of the comprehension task (ToM-15 score 2, up to 15) (Desgranges et al., 2012).

In addition to the above tests, the TREF test was administered to all participants:

• **TREF – Facial Emotion Recognition:** comprises 54 photographs of faces showing six basic emotions: happiness, anger, sadness, fear, disgust, and surprise (Gaudelus et al., 2015). Each emotion is presented with different intensity levels from 20% to 100%. Performance is scored based on the number of correctly recognized emotions from 0 to 54 (Gaudelus et al., 2015). A higher score indicates better facial emotion recognition ability, and a lower score suggests impairments in social cognition, particularly in recognizing and processing others' emotions (Gaudelus et al., 2015). Permission to use the TREF scale was granted by HOGREFE, a scientific publisher. In the current study, the Cronbach alpha value was 0.856.

### 2.6 Statistical analysis

Data analysis was conducted using version 25 of the Statistical Package for the Social Sciences (SPSS) software. The categorical variables were expressed as absolute frequencies and percentages (N, %), while the quantitative variables were expressed as means and standard deviation (SD).

The chi-square (χ^2^) test and Fisher's exact test were used to assess associations between categorical variables. Differences in age between groups were examined using an independent samples *t*-test. To compare cognitive test performance between patients with schizophrenia and healthy participants, a general linear model was used, with cognitive test scores entered as dependent variables and group (patients vs. healthy controls) included as a fixed factor. All analyses were adjusted for relevant covariates, including gender, age, education level, marital status, and family history of psychiatric illness.

An exploratory factor analysis (EFA) using principal axis factoring with oblimin rotation and a tetrachoric correlation matrix was considered for the ToM-15 items using its two structured components, namely the False Belief task and the Comprehension task. No additional or external variables were introduced beyond those derived from the original instrument. Parallel analysis was conducted to determine the number of factors to retain. The EFA approach was appropriate to better understand its dimensionality in this population, as there is currently no validated or consistently replicated factorial structure in the literature, particularly among patients with schizophrenia. The Kaiser–Meyer–Olkin (KMO) measure and Bartlett's test of sphericity were computed to assess sampling adequacy and suitability for factor analysis. The Cronbach's alpha and McDonald's Omega (ω) were calculated to assess its internal consistency.

Confirmatory factor analysis (CFA) was conducted using AMOS version 24 to evaluate the factor structure of the ToM-15 scale. Given the absence of a previously established factorial model, three competing models were specified: (1) a single-factor model, (2) a two-factor model based on false belief and comprehension domains, and (3) a five-factor model derived from the exploratory factor analysis (EFA). Due to sample size constraints, both EFA and CFA were performed on the same sample (*n* = 75). Model fit was assessed using multiple indices, including the chi-square statistic divided by degrees of freedom (χ^2^/df), with values <2 indicating good fit and < 5 considered acceptable. The Root Mean Square Error of Approximation (RMSEA) was interpreted as indicating a close fit when <0.05 and an acceptable fit when <0.11. The Comparative Fit Index (CFI) and Tucker–Lewis Index (TLI) were also examined, with values ≥0.90 indicating acceptable model fit.

Additionally, the normality of the ToM-15 scale was assessed using the Kolmogorov–Smirnov test. The results indicated that the data were not normally distributed; therefore, non-parametric tests were used for the analysis of associations. Correlations between the ToM-15 total score, its subscales, and other scales were examined using Spearman's correlation coefficient. A *P*-value<0.05 was considered statistically significant.

## 3 Results

### 3.1 Sample characteristics

Table 1 shows the sociodemographic and clinical characteristics of patients with schizophrenia and healthy participants. The patient group had a higher proportion of males (60.0%) than females (40.0%), while the healthy participants group had a slightly greater percentage of females (58.0% vs. 42.0% males). This difference was statistically significant (*p* = 0.048). Almost half of the patients (48.0%) had secondary education as compared to 29.2% in the healthy participants group. The proportion of individuals with a university education (37.5%) was significantly higher in the healthy participants group (*p* = 0.017). Marital status of the patients and healthy participants differed significantly (*p* < 0.001). The majority of patients were not married (single, widowed, or divorced), with a proportion 90.7%, while the majority of healthy participants were married (69.4%). In comparison to 12.2% of healthy participants, 35.1% of patients reported a positive family history of psychiatric disorder (*p* = 0.005). The mean age of patients was 54.3 ± 7.3 years, which was considerably higher than that of the healthy participants group (49.7 ± 11.4 years, *p* = 0.016).

**Table 1:** Sociodemographic and clinical characteristics of the total sample (*N* = 125).

	Schizophrenia Patients (*N* = 75)	Healthy participants (*N* = 50)	P-Value	Pearson Chi-Square
	Frequency (%)	Frequency (%)		
**Gender**				
Male	45 (60.0%)	21 (42.0%)	**0.048**	3.900
Female	30 (40.0%)	29 (58.0%)		
**Education level**				8.160
Complementary	27 (36.0%)	16 (33.3%)		
Secondary	36 (48.0%)	14 (29.2%)	**0.017**	
University	12 (16.0%)	18 (37.5%)		
**Marital Status**				
Not Married (single, widowed, divorced) Married	68 (90.7%) 7 (9.3%)	15 (30.6%) 34 (69.4%)	**<0.001**	48.296
**Family History of psychiatric illness**				8.025
Yes	26 (35.1%)	6 (12.2%)	**0.005**	
No	48 (64.9%)	43 (87.8%)		
**Diagnostic (DSM-V)**				
Paranoid	35 (50.7%)			
Disorganized	5 (7.2%)			
Undifferentiated	8 (11.6%)			
Schizoaffective	21 (30.4%)			
	**Mean ±** **SD**	**Mean ±** **SD**		**Effect size d**_**Cohen**_
**Age**	54.3 ± 7.3	49.7 ± 11.4	**0.016**	0.502
**Duration of illness in years**	21.5 ± 9.8			
**Duration of hospitalization in years**	12.3 ± 8.4			
**Number of hospitalizations**	5.4 ± 3.8			

Values in bold are significant at p < 0.05

The distribution of schizophrenia subtypes among the patients was as follows: paranoid (50.7%), disorganized (7.2%), undifferentiated (11.6%), and schizoaffective (30.4%), as determined by the DSM-5 criteria. The average duration of illness was 21.5 ± 9.8 years, with an average number of hospitalizations of 5.4 ± 3.8 and an average duration of hospitalization of 12.3 ± 8.4 years.

### 3.2 Known-groups validity: Comparison between patients and healthy participants

Table 2 shows the comparison between the scales used with patients and healthy participants. Patients with schizophrenia scored significantly lower than healthy participants on the Theory of Mind (ToM-15) measures. In particular, the ToM-15 false belief test score (score 1) was 8.44 ± 0.42 for patients and 13.12 ± 0.55 for healthy participants (*p* < 0.001). The ToM-15 comprehension test score (score 2) was 10.50 ± 0.35 and 13.98 ± 0.46 (p < 0.001, respectively). The total ToM-15 score was 18.94 ± 0.65 and 27.11 ± 0.85 (p < 0.001, respectively). Also, patients exhibited substantially higher SASCCS scores (23.66 ± 1.78 vs. 12.48 ± 2.37, *p* = 0.001) and ACSo scores (11.34 ± 0.87 vs. 6.29 ± 1.20, *p* = 0.003) than healthy participants.

**Table 2:** Comparison of cognitive, social, and emotional measures between patients with schizophrenia and healthy participants.

	Schizophrenia Patients (*N* = 75)	Healthy participants (*N* = 50)	P-Value	Partial Eta Squared
	Mean ± SE	Mean ± SE		
ToM-15 score 1	8.44 ± 0.42	13.12 ± 0.55	**<0.001**	0.238
ToM-15 score 2	10.50 ± 0.35	13.98 ± 0.46	**<0.001**	0.196
Total ToM-15 score	18.94 ± 0.65	27.11 ± 0.85	**<0.001**	0.284
Total SASCCS score	23.66 ± 1.78	12.48 ± 2.37	**0.001**	0.089
Total ACSo score	11.34 ± 0.87	6.29 ± 1.20	**0.003**	0.075
Total QCAE score	89.89 ± 1.99	92.75 ± 2.49	0.431	0.006
Cognitive empathy (QCAE)	55.04 ± 1.35	58.20 ± 1.69	0.202	0.015
Affective empathy (QCAE)	34.84 ± 0.97	34.55 ± 1.22	0.869	0.0002
Total TREF score	23.95 ± 0.86	31.70 ± 1.17	**<0.001**	0.172

**ToM**: Theory of Mind, **ToM-15 score 1:** false belief test score, **ToM-15 score 2:** comprehensive test score, **SASCCS**: The Self-Assessment Scale of Cognitive Complaints in Schizophrenia, **ACSo**: The Self-Assessment of Social Cognition Impairments, **QCAE**: Questionnaire of Cognitive and Affective empathy, **TREF**: Facial Emotion Recognition test.

Values in bold indicate significant values.

SE: Standard Error.

The models were adjusted for gender, age, education level, marital status, and family history of psychiatric illness.

Values in bold are significant at *p* < 0.05.

No significant differences were observed in overall empathy (QCAE total score) between groups (*p* = 0.431), nor in its cognitive (*p* = 0.202) or affective (*p* = 0.869) components.

The TREF emotion recognition task revealed that patients scored substantially lower than healthy participants (23.95 ± 0.86 vs. 31.70 ± 1.17, *p* < 0.001).

### 3.3 Internal consistency

Among patients with schizophrenia, the internal consistency of the total scale measured by Cronbach's alpha coefficient was 0.817 (McDonald's omega = 0.813). For the false belief task, the Cronbach's alpha coefficient was 0.729 (McDonald's omega = 0.723). For the comprehension task, the Cronbach's alpha coefficient was 0.766 (McDonald's omega = 0.772).

### 3.4 Validity of internal structure

A factor analysis was run to test the construct validity of the ToM-15 scale using exploratory factor analysis as the extraction method. All items of the scale could be extracted from the list, and the scale converged on a 5-factor solution using the oblimin rotated matrix with an eigenvalue greater than 1, accounting for 51.8% of the variance (Bartlett sphericity test *p* < 0.001, KMO = 0.430). The observed communalities were generally moderate to high, with an overall range of approximately 0.77. (Table 3)

**Table 3:** Exploratory factor analysis of the TOM-15 scale using oblimin rotation among patients with schizophrenia (*N* = 75).

Oblimin rotated matrix						
Story	Factor 1	Factor 2	Factor 3	Factor 4	Factor 5	Communalities
TOM comprehensive item 15 - The cheese: The cat	0.874					0.802
TOM comprehensive item 14 - The Cheater 0 out of 20	0.739					0.623
TOM comprehensive item 11 - The A boot	0.667					0.677
TOM False belief item 2 - Linda	0.551					0.383
TOM comprehensive item 13 - Dinner at the restaurant	0.515					0.435
TOM comprehensive item 7 - Wet trousers: Automatic sprinkler	0.441					0.563
TOM False belief item 8 - The costume ball	0.437					0.435
TOM False belief item 5 - The ribbons	0.426					0.613
TOM False belief item 12 - Candy	0.424					0.351
TOM False belief item 1 - Chocolate	0.407					0.418
TOM comprehensive item 10 - At the football match	0.380					0.457
TOM False belief item 7 - Wet pants		0.390				0.676
TOM False belief item 15 - The cheese		0.467				0.420
TOM False belief item 6 - The flowers		0.265				0.448
TOM comprehensive item 12 - No candy		0.435				0.288
TOM False belief item 13 - Dinner		0.903				0.904
TOM False belief item 9 - The ball		0.206				0.139
TOM comprehensive item 9 - The ball in the box			0.665			0.705
TOM comprehensive item 3 - The hairdresser Woman on the left			0.453			0.360
TOM comprehensive item 1 - Blue cupboard			0.389			0.440
TOM False belief item 4 - The big boy			0.393			0.283
TOM comprehensive item 2 - Linda has an accident			0.895			0.838
TOM comprehensive item 8 - The Zorro Costume Ball			0.467			0.404
TOM comprehensive item 4 - The tall boy: Boy on the right				0.428		0.283
TOM False belief item 11 - The fisherman				0.516		0.565
TOM False belief item 3 - The hairdresser				0.680		0.612
TOM False belief item 10 - The football match				0.736		0.630
TOM False belief item 14 - The cheater				0.448		0.211
TOM comprehensive item 6 - The flowers: The postman					0.452	0.448
TOM comprehensive item 5 - The ribbons: blue color					0.678	0.647
**Percentage variance explained = 51.8%**	14.5	11.3	10.8	0.08	0.07	
**Kaiser-Meyer-Olkin (KMO)**	0.430					
**Bartlett's test of sphericity**	<0.001					
**Cronbach alpha =** 0.817	0.747	0.513	0.690	0.576	0.559	

Factor 1: Theory of Mind Reasoning; Factor 2: Simple False Belief Processing; Factor 3: Contextual Mental State Integration; Factor 4: Perspective-Taking and Social Interpretation; Factor 5: Deception and Intentionality Understanding.

### 3.5 Confirmatory factor analysis

The confirmatory factor analysis of the TOM-15 test showed that the χ^2^/df ratios ranged from 1.63 to 1.72 across the three models (a single-factor model, a two-factor model based on false belief and comprehension domains, and a five-factor model derived from the exploratory factor analysis (EFA)), indicating an acceptable level of fit. The RMSEA values ranged from 0.089 to 0.099. The CFI and TLI values were low across all models (CFI: 0.360–0.491; TLI: 0.265–0.401) (Table 4).

**Table 4:** Confirmatory factor analysis of the TOM-15 scale.

	χ2	df	x2/df	RMSEA (95% CI)	CFI	TLI
A single-factor model	696.630	405	1.72	0.099 (0.086; 0.111)	0.360	0.265
Two-factor model (false belief and comprehension)	660.131	404	1.63	0.093 (0.080, 0.105)	0.438	0.353
Five-factor model (based on EFA results)	626.999	395	1.72	0.089 (0.076, 0.102)	0.491	0.401

RMSEA: Root Mean Square Error of Approximation; CFI: comparative fit index; TLI: Tucker–Lewis index.

### 3.6 Subscale correlations and convergent validity

The correlations between ToM-15 scores and subscales and the scales used are reported in Table 5.

**Table 5:** Relationships Between TOM-15 Total and Subscale Scores and scales of Social Cognition, Empathy, and Emotion Recognition among patients with schizophrenia.

	ToM-15 Total score	ToM-15 false belief Task	ToM-15 comprehension Task
ToM-15 false belief Task	**0.929**⁎⁎		
ToM-15 comprehension Task	**0.737**⁎⁎	**0.479**⁎⁎	
Cognitive Complaints (SASCCS scale)	**−0.266**⁎	−0.097	**−0.381**⁎⁎
Social Cognition Impairments (ACSo scale)	−0.045	−0.062	−0.041
Cognitive and Affective Empathy (QCAE scale)	0.081	0.075	0.089
Cognitive empathy	0.100	0.103	0.169
Affective empathy	0.095	0.075	0.013
Facial Emotion Recognition Test (TREF)	0.085	0.186	0.042

**ToM**: Theory of Mind**, SASCCS**: The Self-Assessment Scale of Cognitive Complaints in Schizophrenia, **ACSo**: The Self-Assessment of Social Cognition Impairments, **QCAE**: Questionnaire of Cognitive and Affective empathy, **TREF**: Facial Emotion Recognition test.

⁎ p < 0.05, two-tailed, Spearman test.

⁎⁎ *p* ≤ 0.001, two-tailed, Spearman test.

The correlations between the ToM-15 total score and the sub-scores in the patients' sample were high (*r* = 0.929 and *r* = 0.737, respectively; *p* ≤ 0.001), while the correlation between the two sub-scores was moderate (*r* = 0.479; p ≤ 0.001).

Regarding the convergent validity, the ToM-15 total score exhibited a negative correlation with SASCCS score (*r* = −0.266, *p* < 0.05), whereas its association with ACSo, QCAE total score, Cognitive empathy score, affective empathy score, and TREF was not statistically significant. ToM-15 comprehensive test score exhibited a significant negative correlation with SASCCS (*r* = −0.381, *p* ≤ 0.001), while ToM-15 false belief test score did not exhibit any significant associations with any scale.

## 4 Discussion

In this study, the ToM-15 scale was translated into the classical Arabic language and validated among a sample of Lebanese patients with schizophrenia. The validated version demonstrated acceptable internal consistency and preliminary evidence of construct validity, showing that the tool may be useful for the assessment of the social cognitive function in patients with schizophrenia.

A high internal consistency was found (Cronbach's alpha = 0.817). This outcome was similar to that of a study conducted in the same hospital with schizophrenia patients, in which the Cronbach's alpha value for the false belief task was 0.777 and for the comprehension task was 0.794 (Ghosn et al., 2025). Based on the exploratory factor analysis results, a 5-factor structure was found, differing from the original version in which the scale was built with two factors only (false belief test and comprehension test) (Desgranges et al., 2012). However, this disparity may be due to both methodological and cultural variations. The original ToM-15 did not get official psychometric validation, and its structure was established on theoretical rather than statistical reasons. Furthermore, the original study had 175 healthy volunteers of varying ages (20–90 years) and only two levels of education, which may have reduced the factor structure's sensitivity to cognitive and socio-demographic changes (Desgranges et al., 2012). In contrast, the current validation study applied a data-driven approach using Exploratory Factor Analysis to investigate the scale's underlying dimensionality. The finding of five separate characteristics implies that the Arabic version may capture more complex aspects of Theory of Mind performance including False Belief Reasoning, Basic Story Comprehension, Contextual and Causal Comprehension, Visual–Perspective and Detail Comprehension and Deception-Related False Belief Processing. These aspects may be influenced by linguistic, cultural, or educational factors. It is also possible that the increased number of factors reflects a more refined distinction between cognitive processes involved in understanding others' mental states—such as belief attribution, inference, and comprehension of social cues—that were not clearly represented in the original French version. Furthermore, the original scale was administered to healthy participants, but the translated scale was utilized for individuals with schizophrenia, which may elucidate the discrepancies in the results (Desgranges et al., 2012).

KMO values were consistently low across all examined models and this can be attributed to the task-oriented, multidimensional framework of the instrument and the characteristics of the schizophrenic population. The scale consists of interdependent paired story-comprehension tasks and the elimination of items lacks methodological justification, as eliminating individual items necessitates the removal of their corresponding pairs, thus compromising content validity despite satisfactory loadings. Moreover, schizophrenia is distinguished by significant cognitive heterogeneity, fluctuation in symptom-related performance, and the influence of medication, all of which are recognized to diminish inter-item correlations (Wootton et al., 2023). Consequently, low KMO values indicate limited sampling adequacy and, in this situation, may also reflect the clinical and structural characteristics of the measure rather than insufficient construct validity. Therefore, the factor analytic findings of this study must be approached with caution. The employment of tetrachoric correlations and parallel analysis enhances methodological rigor; yet, the comparatively low KMO values and sample size indicate that the stability and generalizability of the derived structure may be constrained.

Given the exploratory nature of the analysis, the 5 factors should not be considered definitive and require confirmation in larger and more diverse samples, and with confirmatory analytic approaches. Moreover, The ToM-15's paired, story-based structure induces interdependence between items, which may impact correlations and results in Exploratory Factor Analysis by combining true latent characteristics with shared task effects. Although this design improves ecological validity, it contradicts certain assumptions regarding item independence in factor analysis. In order to more effectively distinguish general Theory of Mind ability from task-specific variance, future research could implement more sophisticated methodologies, such as multilevel or bifactor models. Nevertheless, these results still offer valuable preliminary insights, albeit with the necessity of circumspect interpretation, on account of the clinical characteristics of the sample and the task-based design of the instrument.

Although the present study provides preliminary evidence supporting the factorial structure of the scale, the sample size should be interpreted with caution in line with contemporary recommendations in factor analysis. As emphasized by MacCallum et al. (1999), sample size adequacy is not determined solely by participant-to-item ratios, but rather depends on characteristics of the data, including communalities and factor loadings (MacCallum et al., 1999). In the current study, most items demonstrated moderate to high communalities and several strong factor loadings, suggesting that the extracted factors capture meaningful shared variance among items. However, the presence of some weaker loadings, together with the relatively modest sample size and low KMO value, indicates that the stability of the factor solution may be limited. Therefore, the identified factor structure should be considered exploratory and preliminary, and future studies with larger and more diverse samples are needed to confirm its robustness and generalizability.

The CFA conducted to evaluate the factorial structure of the ToM-15 scale revealed consistently poor model fit across all tested solutions, including the single-factor, two-factor, and five-factor models. Although the five-factor model derived from the EFA showed relatively better fit indices compared to the alternative models, none of the models achieved acceptable thresholds for CFI and TLI, and RMSEA values remained above recommended cut-offs. Several factors may help explain these findings. First, the relatively small sample size (*n* = 75) may have limited the stability and precision of parameter estimates, particularly given the number of items and complexity of the tested models. Second, both EFA and CFA were conducted on the same dataset, which may have introduced model overfitting and reduced the ability to obtain robust confirmatory evidence. Overall, these results indicate that the dimensional structure of the ToM-15 requires further investigation in larger and independent samples, and highlight the need for additional psychometric refinement before a stable factor solution can be confirmed.

Additionally, our results confirm that patients with schizophrenia exhibit significant impairments in Theory of Mind when compared with healthy participants. The patients' scores on all ToM-15 indices (false belief, comprehension, and total scores) were substantially lower, which replicates robust evidence that ToM dysfunction is a defining characteristic of schizophrenia (Bora et al., 2009). Beyond ToM, patients also showed poorer emotion recognition (TREF). These results are consistent with prior study conducted with patients with schizophrenia in the French population. The study confirmed an impairment in Facial Emotions Recognition in Schizophrenia by demonstrating significant differences between the healthy participants' group and patients' group (Gaudelus et al., 2015). Moreover, patients in our study showed greater self-reported cognitive and social complaints and also these results are consistent with previous studies conducted with patients with schizophrenia in the Lebanese population where these scales were validated (Haddad et al., 2023; Haddad et al., 2021b). The cognitive empathy and affective empathy scores of patients were comparable to those of the healthy participants. However, a previous research has shown reduced cognitive empathy in patients, whereas affective empathy scores are often reported as comparable to, or even higher than, those of control groups. Patients' increased self-reported affective empathy is more likely due to introspective bias, emotional hyper-responsivity, or measurement artifacts than to a genuine increase in empathic ability (Horan et al., 2015).

Regarding the convergent validity, the correlations of ToM-15 with social cognitive complaint, empathy, and TREF were not significant, and only modest correlations were observed with cognitive complaint. Prior researches suggest that enhanced ToM capabilities are linked to enhanced facial emotion recognition, which is not consistent with our findings (Brüne, 2005; Lee et al., 2013). However, a previous study reported a weak or non-existent correlation between social cognitive complaint and objective ToM performance, which aligns with our results (Graux et al., 2019). Patients' self-assessments may be less reliable due to metacognitive or insight deficits, medication or symptom effects, and reduced variability within clinical samples, which could account for this weaker pattern of associations. Additionally, the results may be influenced by the relatively small sample size. It is essential to note that the internal coherence of the instrument and its construct validity as a measure of Theory of Mind are substantiated by the robust correlations observed between the ToM-15 total score and its subscales (false belief and comprehension tests).

Patients with schizophrenia and healthy participants differed across several sociodemographic and clinical measures in the present study, with significant differences observed in numerous variables. The schizophrenia group was primarily male, had a lower level of education, and was less likely to be married than the healthy participants group. This is in line with previous research that has shown that schizophrenia may be more prevalent in men (Li et al., 2022) and frequently results in social withdrawal and challenges in sustaining interpersonal relationships (Green et al., 2015; Fett et al., 2011). The chronic and stable clinical population, which is typical of ToM research on schizophrenia, is indicated by the extended illness duration and higher mean age of patients. Furthermore, the confirmed heritability and familial aggregation of schizophrenia are further supported by the elevated incidence of positive family histories of psychiatric disorders (Chou et al., 2017).

### 4.1 Limitations

Our study has several limitations. The sample size was relatively modest, which may limit the stability and generalizability of the factor structure. Although sample size adequacy in factor analysis depends on data characteristics such as communalities and factor loadings (MacCallum et al., 1999), the presence of some weaker loadings suggests that the findings should be interpreted as exploratory. Future studies with larger and more diverse samples are needed to confirm the robustness of the factor solution. The results' generalizability may be restricted by differences in sociodemographic variables between groups, as well as the inclusion of chronic inpatients recruited from the same institution. Since the population consists of chronically hospitalized patients whose cognitive function might be severely impaired, selection bias is possible. Furthermore, self-reported assessments may have been influenced by deficiencies in insight or metacognitive abilities. Additional selection bias may have resulted from the non-representativeness of the healthy participants group, which was chosen from hospital staff and community volunteers who agreed to participate without being randomly assigned. Information bias is also plausible, given that some participants may have struggled to deliver accurate responses during in-person interviews. Finally, the divergence from the original component structure reveals possible cultural or sample-specific factors that require further evaluation. Also, confirmatory factor analysis could not be performed to validate the derived factor structure because of the relatively small sample size. Future studies with larger samples are needed to confirm these findings. The low KMO values probably indicate limited sampling adequacy and requiring additional examination in future studies. The reliability is assessed only via internal consistency with no evaluation of other important forms such as test-retest reliability and this also should be taken into consideration in futures studies. It is also plausible that residual confounding bias occurred, as unmeasured variables related to social cognitive complaints may have influenced the findings.

## 5 Conclusion

Our findings indicate that the Arabic version of the ToM-15 scale demonstrates acceptable reliability and preliminary evidence of validity for assessing Theory of Mind among Lebanese patients with schizophrenia. Patients had significantly lower levels of ToM, facial emotion detection, and cognitive empathy, as well as higher self-reported social and cognitive complaints, as compared to healthy participants. While these findings support the potential utility of ToM-15 as a tool for assessing theory of mind deficits in Arabic-speaking populations, the findings should be interpreted with caution given the exploratory nature of the analyses and the study's sample characteristics. Further research involving larger and more diverse samples is required to support these findings and investigate cross-cultural applicability.

## CRediT authorship contribution statement

**Maha Bteich:** Writing – original draft, Project administration, Methodology, Formal analysis. **Carole Kassamany:** Writing – original draft, Project administration, Formal analysis. **Chadia Haddad:** Writing – review & editing, Validation, Methodology, Conceptualization. **Hala Sacre:** Writing – review & editing. **Benjamin Calvet:** Writing – review & editing, Resources, Conceptualization. **Pascale Salameh:** Writing – review & editing, Validation, Supervision.

## Funding

This research did not receive any specific grant from funding agencies in the public, commercial, or not-for-profit sectors.

## Declaration of competing interest

I have nothing to declare.
